# Supplementary material for: From Lignocellulosic Residues to Protein Sources: Insights into Biomass Pre-Treatments and Conversion
Source: Polymers (Basel). 2025 Aug 20;17(16):2251. doi: 10.3390/polym17162251 (PMC12389798; doi:10.3390/polym17162251)
Supplement: Supplementary file 1 [file polymers-17-02251-s001.zip › polymers-3773959-supplementary.pdf]

Table S1. Comparative overview of major lignocellulosic biomass pre-treatment methods

Type	Method	Sugar Yield	Process Time	Relative Cost	References
**Chemical**	Dilute Acid Pretreatment	34–49% reducing sugars [69]; up to 90% hemicellulose to xylose [32]	Medium 90 min	Moderate	Tian et al. and Bhatia et al.[32,69]
**Chemical**	Alkaline Pretreatment (NaOH)	High lignin conversion, improves hydrolysis	Medium (1–2 h)	Moderate	Tian et al. and Bhatia et al [32,69]
**Physicochemical**	Steam Explosion	60–80% depending on biomass	Short (5–20 min)	Moderate	Antczak et al., Kululo et al.,[43,73]
**Physicochemical**	Liquid Hot Water	Up to 70% of cellulose conversion	Medium (30–60 min)	Low	Antczak et al., Chen et al. and Kululo et al.[42,43,73]
**Physical**	Microwave Pretreatment	Improves yield when combined with alkaline	Short (10–30 min)	High	Hoang et al. [41]
**Physical**	Ultrasound Pretreatment	Increase of 20–40% in enzymatic hydrolysis	Short (15–60 min)	Moderate	Mankar et al. [45]
**Biological**	Enzymatic Pretreatment	>80% with optimized enzymes	Long (24–72 h)	High	Saratale et al. and Nargotra et al [52,57]
**Biological**	Fungal Pretreatment	Variable, improves cellulose accessibility	Long (days to weeks)	Low	Rodriguez et al., Shankaret al., Suryadi et al., Sajid et al.[50,62,64,66]
**Biological**	Microbial Consortium (CBP)	Efficient conversion with multiple enzymes	Long (3–7 days)	Low to moderate	Sethupathy et al., Cheng, et al., Kuhad, et al.[60,61,68]
**Physicochemical**	Hydrothermal Pretreatment	Moderate sugar and methane yields	Medium (60–120 min)	Moderate	Wang et al., 2024 [132]

Table S2. Substrates and microorganisms used to produce Single-Cell Protein and the respective protein contents obtained in recent years.

	Substrate	Microorganism	Protein content	Reference
**Algae**	Food processing waste	*Chlorella* sp.	15 - 52% (depending on the type of waste)	Putri et al. [135]
	Defatted rice bran	*Chlorella sorokiniana*	16%	Pruksasri et al. [136]
	Reclaiming high-salinity seafood processing sewage	*Chlorella* sp. (MEM25)	52.89 ± 0.65%	Chen et al. [137]
**Microalgae and bacteria**	Agri-industrial wastewaters	*Chlorella vulgaris, Scenedesmu*s, and Purple phototrophic bacteria (PPB)	60%	Hulsen et al. [124]
	Industrial wastewater	*Chlorella sorokiniana* and *Methylococcus capsulatus*	27.62%	Rasouli et al. [123]
	Biogas slurry	*Chlorella vulgaris, Methylophilus, Methylomonas*	>33%	Wang et al. [122]
**Bacteria**	High-ammonia-nitrogen wastewater	Hydrogen-oxidizing bacteria (HOB): *Paracoccus denitrificans* (Y5) and *P. versutus* (D6)	67.34–73.73%	Dou et al. [138]
	Urban biowaste (vegetable food waste, animal food waste, yard waste and flowers, paper and cardboard containers, and other biodegradable and non-biodegradable materials	Mixed culture enriched in *Methylococcales* and *Methylophilales*	8 e 20%	Khoshnevisan et al. [139]
	Swine manure, chicken manure, cow manure, and buffalo manure	*Rhodopseudomonas faecalis* (PA2)	62.70%	Patthawaro and Saejung [140]
	Wastewater	Mixed methanotrophic culture	>41%	Zha et al. [115]
	Fruit and Vegetable Waste	*Lactobacillus* sp.	0.4834	Kaur and Chavan [114]
	Fruit juice industry wastewater	Purple non-sulfur bacteria (PNSB)	>40%	Rashid et al. [141]
**Fungi**	Sugarcane	*Aspergillus niger*	450mg/100ml	Kumari et al. [142]
	Water hyacinth	*Rhodosporidium toruloides* (NCIM 3547)	53.60 g/g	Alankar et al. [143]
	Fiber sludge (paper industry waste)	*Pleurotus ostreatus* (LGAM 1123)	44.8 ± 0.8%	Bakratsas et al. [144]
	Food waste (Fresh pineapple, orange, banana, sugarcane, and garlic peels)	*Aspergillus niger*<br>*Penicillium citrinum*<br>*Penicillium custom*	9.79 ± 0.11 g/L<br>9.41 ± 0.15 g/L<br>7.75 ± 0.11 g/L	Ahmed et al. [88]
**Yeast**	Brown Seaweed and Spruce Wood	*Candida utilis*	43-53%	Sharma et al. [102]
	Corncob hydrolysate and urea	*Candida intermedia*	484.2 g kg^{-1}	Wu et al. [85]
	Food waste	*Saccharomyces cerevisiae*	39.80%	Gervasi et al. [145]
	Rice straw	*Geotrichum candidum* and *Pichia kudriavzevii*	*G. candidum* 48.66% and *P. kud* 37.11%	Diwan and Gupta [103]
	Rye straw, Rye bran, Oat bran	*Yarrowia lipolytica*	30.5–44.5%	Drzymała et al. [99]

Spruce wood (+enzymatic hydrolysates of poultry by-products and urea)	*Cyberlindnera jadinii, Wickerhamomyces anomalus Blastobotrys adeninivorans*	47-51%	Lapeña et al. [101]
Fresh sugar beets	*Saccharomyces cerevisiae*	47.78%	Razzaq et al. [146]
Sugarcane Bagasse	*Spathaspora passalidarum*	18.72 g/L	Bonan et al. [105]
Orange peels, apple waste, and sugar cane molasses	*Saccharomyces cerevisiae*	44 - 56% (depends on the type of waste)	Khan et al. [147]
Rice straw	*Saccharomyces cerevisiae*	51%	Yang et al. [104]
Biogas slurry	*Nectaromyces rattus*	35.96%	Zhang et al. [148]
Effluent of candies production and digestate from agricultural digesters	*Saccharomyces cerevisiae*	28% w/w	Bertasini et al. [149]
Orange peels	*Candida utilis*	6.22%	Carranza-Méndez et al. [150]
Wasted date molasses	*Hanseniaspora guilliermondii* (JQ690237); *Hanseniaspora uvarum* (JQ690236); *Issatchenkia orientalis* (JQ690240); *Cyberlindnera fabianii* (JQ690242).	54.30%	Hashem et al. [151]
Wastewater from *Coffea arabica*	*Candida sorboxylosa*	38%	Pillaca-Pullo et al. [152]
Multi Food-Waste Substrate (Fish wastes, represented by head, viscera, skin and bones, pineapple, banana, apple, and citrus peels).	*Saccharomyces cerevisiae* (ATCC 36858)	40.19%	Tropea et al. [11]
Waste organic acids and nitrogen	*Saccharomyces cerevisiae (DSM 70424)*	0.94 g/L	Zeng et al. [153]
Fruit wastes	*Saccharomyces cerevisiae*	29-45% (depends on the type of waste)	Abodunde and Akin-Osanaiye [154]
Starch processing wastewater	Co-cultivation of yeasts (*Candida utilis, Candida tropicalis, Saccharomycopsis fibuligera, Saccharomyces cerevisiae* and *Geotrichum candidum)*	3.06 g/L	Tian et al. [155]
Wheat straw and ammonia sulfate	*Trichosporon cutaneum* (MP11)	36-37%	Zhang et al. [156]
Water hyacinth (aquatic weed)	*Saccharomyces cerevisiae*	38.43%	Saravanan et al. [113]
Biogas slurry	*Debaryomyces hansenii*	22.68%	Liu et al. [157]
